# Automatic migraine classification using artificial neural networks

**DOI:** 10.12688/f1000research.23181.2

**Published:** 2020-07-17

**Authors:** Paola A. Sanchez-Sanchez, José Rafael García-González, Juan Manuel Rúa Ascar

**Affiliations:** 1 School of Engineering, Universidad Simón Bolívar, Barranquilla, Atlántico, 00000, Colombia

**Keywords:** artificial neural networks, migraine, supervised learning, automatic classification techniques

## Abstract

**Background**: Previous studies of migraine classification have focused on the analysis of brain waves, leading to the development of complex tests that are not accessible to the majority of the population. In the early stages of this pathology, patients tend to go to the emergency services or outpatient department, where timely identification largely depends on the expertise of the physician and continuous monitoring of the patient. However, owing to the lack of time to make a proper diagnosis or the inexperience of the physician, migraines are often misdiagnosed either because they are wrongly classified or because the disease severity is underestimated or disparaged. Both cases can lead to inappropriate, unnecessary, or imprecise therapies, which can result in damage to patients’ health.

**Methods:** This study focuses on designing and testing an early classification system capable of distinguishing between seven types of migraines based on the patient’s symptoms. The methodology proposed comprises four steps: data collection based on symptoms and diagnosis by the treating physician, selection of the most relevant variables, use of artificial neural network models for automatic classification, and selection of the best model based on the accuracy and precision of the diagnosis.

**Results:** The artificial neural network models used provide an excellent classification performance, with accuracy and precision levels >97% and which exceed the classifications made using other model, such as logistic regression, support vector machines, nearest neighbor, and decision trees.

**Conclusions:** The implementation of migraine classification through artificial neural networks is a powerful tool that reduces the time to obtain accurate, reliable, and timely clinical diagnoses.

## Introduction

Cephalalgia or headache represents one of the most common types of pain experienced by humans. Headaches usually occur intermittently. The most frequent forms correspond to migraine and tension headache. Migraines are classified as chronic disorders of the nervous system and are characterized by the onset of recurrent symptoms or episodes associated with headache, which can range from moderate to severe pain and includes throbbing or vibrating pain; furthermore, migraines can be experienced unilaterally or bilaterally and can trigger other symptoms, such as nausea, vomiting, weakness, and light and sound sensitivity ( Charles, 2013; Deza, 2010; IHS, 2018).

Both chronic and recurrent/relapsing headaches can cause pain and distress, but they rarely reflect a serious health problem. However, any change in the pattern or nature of the headache could be a sign of a serious complication, i.e., change in pain frequency from sporadic to frequent or pain severity from mild to acute; hence, medical attention should be sought as soon as possible ( Goadsby *et al.*, 2002).

Although headache is generally a benign and transitory disorder that in most cases ceases spontaneously or with the aid of analgesics, it can also be caused by a serious life-threatening illness such as meningitis, brain tumor, hypercholesterolemia, heart problems, or subarachnoid hemorrhage (arteriovenous malformation). On the other hand, certain types of headaches, such as migraines, although benign, cause much suffering in affected individuals and represent an economic burden because of the high number of work-loss hours they cause ( Trillos, 2010).

In 1988, the Classification Committee of the International Headache Society ( IHS, 2018) published the current classification of headache types, which divides headaches into primary and secondary headaches. Primary headaches include migraines, tension-type headaches, paroxysmal headaches (cluster headaches and paroxysmal hemicrania), and benign miscellaneous headaches. Secondary headaches are those caused by vascular disease, infection, tumors, alteration in cerebrospinal fluid production, cranial trauma, neuralgia, etc.

Discrimination among migraines with and without aura and other types of migraines and headaches is established based on the specific criteria established by the International Headache Society ( Charles, 2018; IHS, 2018; Rasmussen & Olesen, 1992; Viana *et al.*, 2017). To diagnose migraines, the patient’s medical history and symptoms are assessed and a physical and neurological examination is performed; these are sometimes accompanied by specialized examinations such as magnetic resonance imaging, tomography, electroencephalogram, and lumbar puncture ( Evans, 2019). An important part of diagnosing migraines is discarding other medical conditions that could be causing the symptoms ( Altintop *et al*., 2017; Diamond *et al*., 2007; Giffin *et al.*, 2003; Goadsby & Holland, 2019; Karsan & Goadsby, 2018; Maniyar *et al*., 2015).

Isaza *et al.* (1997) conducted a statistical study in mid-1981 and 1989 in Colombia and showed that 11.6% of women and 3.4% of men suffered from migraines. Similarly, Ramírez & Urrea (2012) reported that in 1997, of 3,401 patients assessed in Colombia in the outpatient department of the neurology service, 848 (24.93%) were due to primary headache, which is an important reason for outpatient consultation. Migraines occurred in 617 (18.14%) patients, with aura in 255 (7.5%) and no aura in 362 (10.64%).

Previous studies on migraine classification focused on the neurological or genetic aspects of the disease, leading to investigations that allowed for the classification of various types of migraines based on the study of encephalograms ( Akben *et al.*, 2012; Akben *et al.*, 2010; Akben *et al.*, 2016; Alkan & Akben, 2011; Altintop *et al*., 2017; Bellottia *et al*., 2007; Martins-Oliveira *et al*., 2017; Subasi *et al.*, 2018), signals emitted by body temperature sensors, blood oxygen, heart rate, and electrodermal activity data ( Chong *et al*., 2017; Koskimäki *et al*., 2017; Schwedt, 2013), or genetic analysis ( Gormley *et al*., 2016). Although these studies attempted to classify migraines with precision levels of >70%, the methods used required a direct measurement of the variables using medical devices connected to the patient, which can cause variation in the data; lead to long waiting times for the appointment of specialized examinations, particularly in Latin American countries; or result in a lack of availability of medical equipment in rural regions. As stated previously ( Alkan & Akben, 2011), numerous investigations have attempted to develop automatic diagnostic methods for migraines. However, no definitive diagnostic method for migraines has yet been accepted by the authorities on the subject (IHS).

Currently, there is a recurrent problem in the diagnosis and treatment of migraines, which includes, among others, the following needs: (i) proper reading and identification of the patient’s primary and secondary symptoms, (ii) precise and timely identification of the type of migraine, (iii) continuous monitoring of the symptoms, and (iv) adequate treatment. In the early stages of the pathology, patients visit the emergency services or outpatient consultation departments, where timely identification largely depends on the expertise of the treating physician and the continuous monitoring of the patient ( Burch *et al.*, 2018; Burch, 2019; Deza, 2010; Evans & Johnston, 2011; Trillos, 2010; Wang *et al*., 2019). However, owing to the scarcity of time to establish a diagnosis, the inexperience of the physician, or shortcomings in the patient–physician communication of symptoms, the pathology is often misdiagnosed or the severity of the disease is underestimated, leading to inappropriate, unnecessary, or imprecise therapies, which can result in complex damages to patients’ health ( Evans & Johnston, 2011). Migraines can be misdiagnosed as tension headache, sinus headache, or other types of headache. A diagnosis of migraine should be considered when there are recurrent and debiliting headaches without secondary warning signs ( Burch *et al.*, 2018; Burch, 2019; Wang *et al*., 2019). Therefore, misdiagnosis or incorrect classification of the type of migraine and inadequate treatment of the pathology constitute the underlying problem associated with migraines ( Deza, 2010; Trillos, 2010).

The need for tools aimed at enabling appropriate decision-making has led to an accelerated interest in the development of data classification models in recent decades, which is especially intended to overcome the theoretical, conceptual, and practical limitations of many of the techniques currently available. This has resulted in the emergence of a wide range of models, among which artificial neural networks have demonstrated high potential given their adaptability, generalizability, and learning capabilities and because of the possibility of representing nonlinear relationships ( Sánchez-Sánchez & García-González, 2017). Artificial neural networks and their recent architectures, such as deep neural networks, have been used effectively in data classification tasks and display better results than other techniques, such as logistic regression, decision trees, Bayesian classifiers, etc.; their strength lies in their high capacity to dynamically create complex prediction functions and emulate human learning ( Nikam, 2015; Sánchez-Sánchez & García-González, 2017; Sánchez-Sánchez & García-González, 2018).

This article seeks to contribute to the early identification of different types of migraines through the use of supervised learning techniques based on artificial intelligence, which allow overcoming the difficulties encountered. The purpose of this study is to develop a classification model that allows the determination of the type of migraine a patient suffers based on the analysis of its symptoms and medical history. The novelty, significance, and relevance of this study are outlined as follows:

• An indirect method aimed at classifying the type of migraine experienced by a patient, which, unlike existing methodologies, does not use procedures requiring brain wave measurement or the use of sensors.

• The systematic migraine classification process used includes the stages of data collection based on symptoms and diagnosis by the treating physician, selection of the most relevant features, use of different classification models, and selection of the most suitable model based on the accuracy and precision of diagnosis.

## Methods

The strategy developed here is based on a systemic approach oriented to the specification of artificial neural network models for the classification of migraines with and without aura, highlighting the relevance of considering key aspects that lead to strong implications in their application, such as the selection of variables and the performance measures that allow for the selection of the best model.

**Figure 1.**
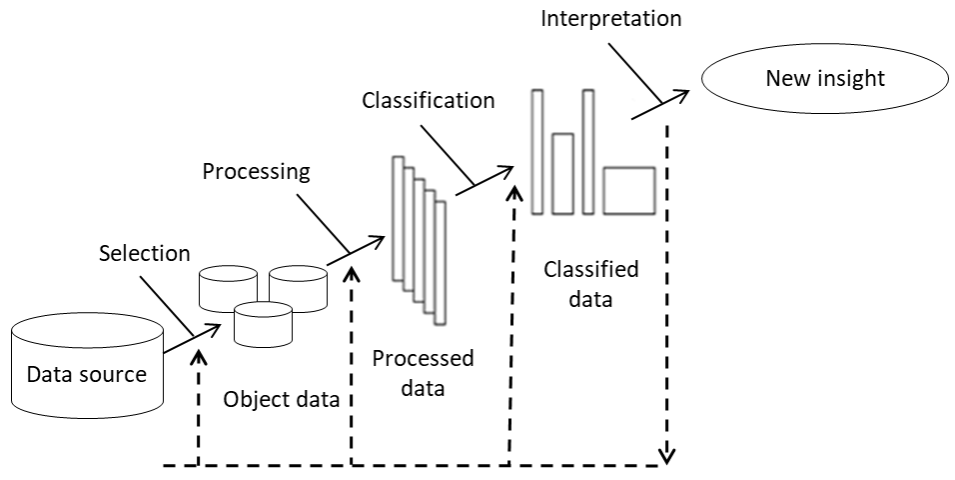
Flow diagram for the classification of patients with migraine.

Note: It is important to understand that the proposed methodology does not include the data collection process. In the current research, a retrospective database is used, to which the proposed methodology is applied. The results section describes the data population and the selection and exclusion criteria.

Starting from the existence of a data source that includes various typical symptoms of patients with migraines, Figure 1 outlines the steps in the classification of patients with migraines and the comparison model ( García-González *et al*., 2019; Sánchez-Sánchez *et al.*, 2019b).

The elements included in Figure 1 are discussed below.

1. Selection: The data selection phase is directed toward the preliminary analysis of the various data sources (if any), their features, and aspects related to the environment in which they are obtained. This selection can be defined as a process of approximation to the available information through subjective analysis and statistical treatment in order to infer the hidden structure of data.

Knowing the goals and the data that will enable this process are key factors for a successful selection process.

2. Processing: Data processing consists of analyzing and transforming the input variables with the aim of minimizing noise, highlighting important relationships, and detecting errors to enable the recognition of hidden patterns. Processing comprises four types of processes: the first aimed at minimizing noise via the transformation of the object data and the elimination of irregular patterns (atypical data; poor typing; blank, incomplete, and inconsistent data, etc.); the second aimed at scaling the large-sized object data; the third aimed at considering the syntactic transformations of the object data and facilitating its handling, without leading to changes in the results; and the fourth aimed at the selection of the variables, characteristics, or attributes that will be taken into account. This selection largely depends on the knowledge that the data modeler has on the data sources, and it is his/her task to decide whether to include each variable in the model following some previously established criteria. Typically, not all potential variables are equally informative as they may be correlated, present noise, or have no meaningful relationship with the classification ( Londoño González & Sánchez, 2015)

3. Classification: Classification involves searching patterns of interest that express dependency on the data and allows groups with similar features to be established. The process essentially consists of assigning each individual (entity or data) its own category or class, thereby creating sets of individuals sharing some feature that differentiate them from the rest. Classes can be binary, Yes or No, or multiclass, which include more than two categories. At this stage, machine learning, whose objective is to develop techniques that allow computers to learn, is used.

The past few years have seen the proliferation of different automatic classification techniques based in learning machine, including artificial neural networks, decision trees, logistic regression, Bayesian classifiers, nearest neighbor, support vector machines (SVMs), and multiple discriminant analysis ( Doupe *et al.*, 2019; Waring *et al.*, 2020).

**Figure 2.**
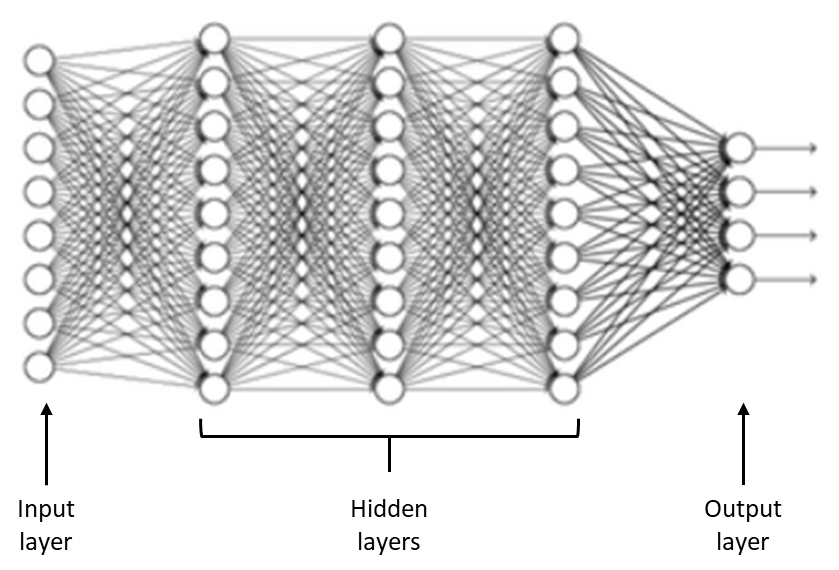
General scheme of an artificial neural network.

In this article, because of the robustness of the data management technique, adaptability, and acknowledged generalization capacity, artificial neural networks are used to classify patients with migraines. Artificial neural network is represented as a three-layer model: an input layer, one or more hidden layers, and an output layer ( Figure 2) ( Sánchez-Sánchez *et al*., 2020b). An input layer represents the variables that influence the model, hidden layers perform the processing, and the output layer corresponds to the various migraine classes.

Logistic regression models, SVMs, nearest neighbor, and decision trees are also implemented in order to compare data collected using other classification techniques.

4. Interpretation: In this phase, evaluation of the quality of the model is performed by analyzing and comparing the results from different metrics used for the classification based on the data obtained in the classification stage, which is aimed at understanding the main characteristics of the model.

For classification problems, common performance metrics are as follows:

▪ Accuracy: Proportion of correctly classified instances.

▪ Precision: Also called positive predictive value, it represents the fraction of correctly predicted positives among those classified as positive.

## Results

Method: We used the proposed methodology for the classification of migraines.

Population: This study used a database comprising 400 retrospective medical records of users diagnosed with various pathologies associated with migraines. Data was compiled in the research of the master's thesis “Analysis of Artificial Neural Network Models, for a Migraine Diagnosis System with Aura and without Aura” ( De la Hoz *et al.*, 2014). Data were recorded by trained medical personnel at the Hospital Materno Infantil de Soledad during the first quarter of 2013. The compiled database contains information regarding patient identification, healthcare provider identification, treating physician identification, symptoms, diagnosis, and treatment. However, this study only uses symptoms and diagnosis. No patient identifiable data was required. See underlying data ( Sanchez-Sanchez *et al.*, 2020a).

As inclusion criteria, we have adult users diagnosed with migraine-associated pathologies. Users with other pathologies are excluded.

Procedure: Selection and processing: Based on compiled database, tasks related to noise elimination, error detection, and data translation into numerical variables were performed.

Variables that influence the identification of the type of migraine were selected, focusing on symptoms and diagnosis and disregarding identification and treatment variables. This led to a selection of 23 variables associated with the symptoms or signs that a patient may present, and 1 variable associated with the diagnosis that allows the identification of the type of migraine. Table 1 presents a list of the 24 identified variables and their description.

**Table 1.** List of identified variables.

	Description	Name		Description	Name
1	Patient’s age	Age	13	Lack of speech coordination	Dysphasia
2	duration of last episode in days	Duration	14	Disarticulated sounds and words	Dysarthria
3	Frequency of episodes per month	Frequency	15	Dizziness	Vertigo
4	Unilateral or bilateral pain location	Location	16	Ringing in the ears	Tinnitus
5	Throbbing or constant pain	Character	17	Hearing loss	Hypoacusis
6	Pain intensity, i.e., mild, medium, or severe	Intensity	18	Double vision	Diplopia
7	Nauseous feeling	Nausea	19	Simultaneous frontal eye field and nasal field defect and in both eyes	Visual defect
8	Vomiting	Vomit	20	Lack of muscle control	Ataxia
9	Noise sensitivity	Phonophobia	21	Jeopardized conscience	Conscience
10	Light sensitivity	Photophobia	22	Simultaneous bilateral paresthesia	Paresthesia
11	Reversible visual symptoms	Visual	23	Family background	Family
12	Reversible sensory symptoms	Sensory	24	Diagnosis of migraine type	Type

The type of variable states the different values that it can assume and can be either continuous, e.g., age, or binary, e.g., nausea. The variable “Type” indicates the diagnosis issued by the treating physician based on the symptoms and medical record of the patient, with the possibility of presenting of one of the following classifications:

1. Typical aura with migraine

2. Migraine without aura

3. Typical aura without migraine

4. Familial hemiplegic migraine

5. Sporadic hemiplegic migraine

6. Basilar-type aura

7. Other

**Figure 3.**
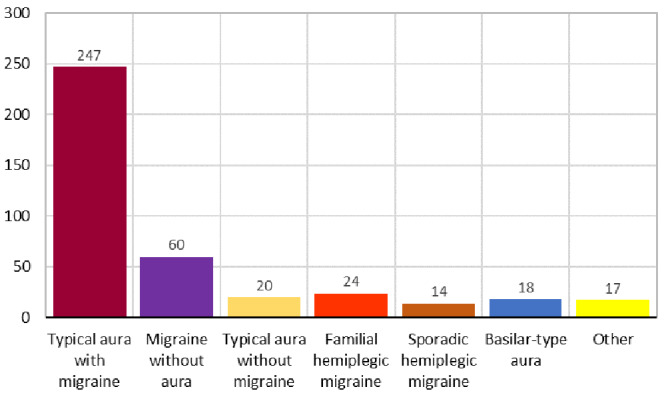
Number of cases by type of migraine.

Figure 3 presents the distribution of cases according to the classification of migraine types carried out in the study and which serves as a criterion for verifying accuracy and precision.

Tests are conducted with the complete dataset (23 variables), and variable reduction, recursive elimination of variables, and selection of the best number through cross-validation is applied, the latter in order to eliminate variables with redundant information. This produces a reduced set of 18 variables.

Classification: Preprocessed data are used as inputs to five different classification models: a multilayer perceptron-type artificial neural network (MLP), which is validated using different network configurations that differ in the number of neurons and hidden layers; a logistic regression model; an SVM model; a nearest neighbor model; and an optimized classification and regression tree (CART).

Python 3 script is used with Scikit-learn 0.23.1 ^1^ and pandas’ 1.0.4 libraries ( Sanchez-Sanchez *et al.*, 2020a).

The artificial neural network model used is equivalent to an MLP trained using backpropagation. The configuration parameters used for the artificial neural network models are as follows:

• Input neurons: 23 variables corresponding to the first 23 variables presented in Table 1 for the complete model and 18 variables for the reduced model.

• Hidden neurons: An iterative optimization process is performed on each hidden layer, varying from 1 to 25. The number of neurons per layer is based on the best accuracy obtained.

• Hidden layers: An incremental construction process of 1 to 4 layers is conducted. The best number of neurons from the previous hidden layer is taken and is iteratively increased from 1 to 25 in the new hidden layer.

• Output neurons: Seven neurons that correspond to each class of migraine

• Transfer function: Logistics

• Performance metrics: Accuracy and precision

• Learning algorithm: Adam ^2^

• Number of epochs: 2000

Logistic regression uses regularization with built-in cross-validation, which automatically selects the best hyperparameters for model fit. A 10-fold and random restart model was used ^3^.

The SVM model uses the SVC function for classification, in which the fit time quadratically scales with the number of samples ^4^.

In the nearest neighbor model, the optimal number of neighbors is estimated based on the accuracy, and the Euclidean function with uniform weights is used as a distance measure.

The decision tree model included in the Scikit-learn library corresponds to an optimized version of CART (Classification and Regression Trees) that uses discrete numerical variables and where an iterative process is used to fit the number of levels based on accuracy.

In all cases, the dataset is divided into two sets: training and testing, the first corresponding to 80% of the data (320) and the second to 20% (80).

### Discussion

Results were validated based on the type of migraine diagnosed by the treating physician and using the respective measurement made during the classification process provided by the artificial neural network.

Table 2 presents the results of the performance measures for various artificial neural network configurations and classification models. Precision, a metric calculated independently for each of the classes, is taken as the weighted average of the seven migraine classes.

**Table 2.** Results of the performance metrics of various artificial neural network configurations and classification models.

Complete model with 23 variables			
Hidden Layers	Internal configuration (number of hidden neurons)	Accuracy	Precision
1	10	**0.975**	**0.97**
2	(10, 15)	0.9625	0.97
3	(10, 15, 15)	0.9375	0.95
4	(10, 15, 15, 25)	0.9375	0.94
Logistic regression		0,875	0.9563
Support vector machines		0.8625	0.9531
Nearest neighbor		0.7875	0.8719
Decision trees		0.8125	0.8100
Model reduced to 18 variables			
Hidden Layers	Internal configuration (number of hidden neurons)	Accuracy	Precision
1	20	**0.975**	**0.98**
2	(20, 25)	0.95	0.95
3	(20, 25, 25)	0.9375	0.92
4	(20, 25, 25, 20)	0.85	0.79
Logistic regression		0,925	0.9467
Support vector machines		0.85	0.8844
Nearest neighbor		0,825	0.8594
Decision trees		0.8578	0.8650

The results presented in Table 2 indicate the following results:

• The maximum accuracy for both 23 and 18 variables is obtained by using a artificial neural network model with 10 hidden neurons, which results in an accuracy of 97.5%. This means that the classification of migraines by the artificial neural network coincides with that issued by the treating physician in 97% of the 80 cases comprising the test set.

• The artificial neural network models show accuracies and precisions >90%, highlighting values obtained with the artificial neural network model with 10 hidden neurons and a hidden layer for 23 variables and 20 hidden neurons for 18 variables, which reaches values >97% in both metrics, thereby indicating adequate classification.

• Those models with all variables have accuracies >80%, with the exception of the nearest neighbor model, which strongly demonstrates that the predicted values coincide with the real values, allowing for the correct classification of the different types of migraine in percentages >80%.

• The maximum average weighted precision was obtained with the artificial neural network model with a layer of 20 hidden neurons that was reduced to 18 variables, which obtained a value of 98%; this indicates that there is a 98% probability that the model classifies the migraine within a certain type and that the treating physician has also classified it as such.

• The precisions obtained using logistic regression and SVM models do not differ greatly from the value obtained using artificial neural networks, even exceeding them in complex artificial neural network configurations with three and four hidden layers.

• Logistic regression, nearest neighbor, and decision tree models show better accuracy values when using models reduced to 18 variables.

• The values of accuracy and precision obtained using artificial neural network models do not favor the increase of hidden layers, leading to reduction phenomena in both metrics as the number of hidden layers and neurons increases, which is representative of overlearning processes.

The experiment results indicate that the performance of the proposed method yields satisfactory results and outperforms machine learning algorithms in migraine classification with a large gap in terms of accuracy and precision. The proposed method is generic as it does not need handcrafted features and can be easily adapted to different detection tasks, requiring minimal pre-processing. The strategy proposed has successfully transferred knowledge from the source to the target domain despite the limited dataset size. During the proposed approach, we observed that no over-fitting occurs to impact the classification accuracy adversely.

Some aspects that justify and favor the development of artificial neural network models for data classification are as follows ( Sánchez-Sánchez *et al.*, 2019a): 

1. The data generating process is often unknown and difficult to identify, thereby limiting the capacity of parametric models to appropriately classify data. Artificial neural networks are self-adaptive models that do not require *a priori* assumptions about the problem under study, a highly desirable feature in cases in which the data generating mechanism is unknown ( Qi & Zhang, 2001).

2. Real data often show unstable behavior. The ability of the artificial neural network to learn and be generalized allows the model to learn complex behaviors directly from the data and correctly infer the unseen part of the data from the acquired knowledge ( De Gooijer & Kumar, 1992).

3. The relationships between the data and the variables that explain its behavior are complex. The universal approximation characteristics of artificial neural networks allow them to identify hidden dependencies, especially nonlinear dependencies ( Cybenko, 1989; Franses & Van Dijk, 2000; Hornik, 1991; Hornik *et al.*, 1989), thereby favoring the representation of complex relationships.

4. Data units can be very large or very small. Artificial neural networks are flexible in relation with the values they receive and deliver and do not require prior treatment.

5. Data are obtained from multiple fields of knowledge. Their functional flexibility and characteristics as universal approximators allow artificial neural networks to represent complex behaviors regardless of the field of knowledge those data belong to.

### Comparison with previous studies

For the sake of comparison, the performance of the classification obtained by the best model proposed here is compared with the results of the previously published work for classification of migraine in Table 3.

**Table 3.** Comparison of precision for migraine classifications reported in previous studies.

Reference study	Classification model	Precision
Migraine diagnosis support system based on classifier ensemble ( Jackowski *et al*., 2014)	LAD Tree	75.9%
Automatic diagnosis of primary headaches by machine learning methods ( Krawczyk *et al*., 2013)	Random Forest	81%
Analysis of repetitive flash stimulation frequencies and record periods to detect migraine using artificial neural network ( Akben *et al.*, 2012)	ANN	83.3%
Classification of multi-channel EEG signals for migraine detection ( Akben *et al.*, 2016)	SVM	85%
Effect of photic stimulation for migraine detection using random forest and discrete wavelet transform ( Subasi *et al*., 2019)	Random Forest	85.95%
Analysis of Artificial Neural Networks Models, for a System of Diagnoses of Migraines with Aura and without Aura ( De la Hoz & Rúa *et al.*, 2014)	ANN	91.04%
A clinical decision support system for the diagnosis of probable migraine and probable tension-type headache based on case-based reasoning ( Yin *et al*., 2015)	CBR	93.14%
This study	ANN (complete)	97%
This study	ANN (reduced)	98%

LAD - least absolute deviations, ANN – artificial neural network, SVM – support vector machine, CBR – case-based reasoning

Table 3 presents the migraine classification results from previous studies using precision as a performance measure. The proposed artificial neural network model with 10 neurons (97% precision with all variables and 98% precision with reduction to 18 variables) achieved a better performance compared to the methods in ( Akben *et al.*, 2012; Akben *et al.*, 2016; De la Hoz & Rúa *et al.*, 2014; Jackowski *et al*., 2014; Krawczyk *et al*., 2013; Subasi *et al*., 2019; and Yin *et al*., 2015) with precisions 75.9%, 81%, 83.3%, 85%, 85.95%, 91.04% and 93.14%, respectively..

## Conclusions

This study presents the development of a methodology for migraine classification using artificial neural network models. The results show that artificial neural networks can achieve higher precision and accuracy than other classification models commonly used in machine learning, which is consistent with the results found when compared with various models proposed in the literature. The first experiments included 24 variables involved in migraine diagnosis, achieving a 97% precision level for the artificial neural network model. However, a second testing phase reduced the set of variables to 18, reaching a precision of 98%. This not only proves that the artificial neural network model is effective for the proper classification of the different types of migraine but shows that it can also be improved by considering a reduced set of variables that significantly affect the classification.

The implementation of migraine classification through artificial neural networks is a powerful tool whose potential has only incipiently been developed and which constitutes a valuable preliminary progress on the broad problem that automatic detection of migraines can encompass. The significance of this work lies in proposing an accurate and timely method of migraine classification that may support the diagnosis established by the treating physician based on an appropriate reading and identification of the primary and secondary symptoms that the patient presents and that results in the appropriate choice of treatment.

The main novelties are as follows:

• The development of a holistic methodology for migraine classification that encompasses selection of correct data and interpretation of the results obtained.

• The successful use of artificial neural network models for the classification of different types of migraines based on patients’ symptoms, which use a database with data from patients who have experienced migraines and have been diagnosed by their treating physicians, resulting in a model that allows for the near-perfect distinction among the different types of migraine.

In addition, increasing the number of patient records in the database can lead to more accurate results in migraine classification because it enhances learning. Future research may be oriented to the comparison of different deep neural network architectures and the analysis of the treating physician's diagnosis versus the theoretical classification of the types of migraine.

## Data availability

### Underlying data

Code Ocean: Migraine Classification Model. https://doi.org/10.24433/CO.2826453.v1 ( Sanchez-Sanchez *et al.*, 2020a)

This project contains the following underlying data:

- Migraine.cvs (Dataset contain medical records of patient with migraines)
- Migraine Dataset Description.txt (Description of data.)

### Reporting guidelines

Zenodo: STARD checklist for ‘Automatic Migraine Classification Using Artificial Neural Networks’

http://doi.org/10.5281/zenodo.3872279 ( Sanchez-Sanchez, 2020)

Data are available under the terms of the Creative Commons Attribution 4.0 International license (CC-BY 4.0).

### Software availability

Code for the model is available from Code Ocean: https://doi.org/10.24433/CO.2826453.v1 (code.ipynb)

License: GNU General Public License (GPL)

## Ethical considerations

### Ethical approvals

Data used in the recent study are the result of the master's thesis work of the author Juan Manuel Rúa Áscar, who had authorization for their academic use by the Hospital Materno Infantil de Soledad, in accordance with Colombian Law 1581 of 2012 and Decree 1377 of 2013 art. 10, which regulates the treatment of personal information and allows the use for scientific purposes, and that authorization is it extends for the current study.

Ethics approval for the use of the original data in the current study was obtained from Universidad Simón Bolívar Research Ethics Committee on 12 May 2020 (reference PRO-CEI-USB-CE-0328-00)

## Consent

This research is not a clinical trial and doesn’t involve any direct patient contact. Anonymized retrospective data collected as part of routine clinical care are included. As a retrospective patient records study, consent was not requested from individual patients. In such cases the ICO code of practice states that explicit consent is not generally required.

## Footnotes

^1^Scikit-learn (formerly scikits.learn and also known as sklearn) is a free software machine learning library for the Python programming language. https://scikit-learn.org

^2^ arXiv:1412.6980

^3^ https://scikit-learn.org/stable/modules/linear_model.html#logistic-regression

^4^ https://scikit-learn.org/stable/modules/generated/sklearn.svm.SVC.html#sklearn.svm.SVC
