# Supplementary material for: Automatic migraine classification using artificial neural networks
Source: F1000Res. 2020 Jul 17;9:618. Originally published 2020 Jun 16. [Version 2] doi: 10.12688/f1000research.23181.2 (PMC8564744; doi:10.12688/f1000research.23181.2)
Supplement: Peer review history

# Peer review: Reviewer response for version 2

**DOI:** 10.5256/f1000research.28066.r95716

**Published:** 2021-11-02

**Authors:** Stelmo Magalhães Barros Netto

**Affiliations:** 1 Federal University of Maranhão, São Luís, Brazil

The paper is about migraine classification from neural networks and patients' symptoms. In general, the present work is well written and the results are encouraging.

 However, the population of 400 records is not enough for training a neural network, and only a part of this data is used for training. In addition, the contributions of these works are unclear, as there are many others performing the same task and using the same methods (see Trojan Fenerich *et al*., 2020 ^1^). Thus, a larger dataset, originating from various sources, is required to not include data bias when obtaining the results.

 The following points can improve the methodology and results of this work:

- The method used to select the variables is not clear enough due to several models being used in addition to neural networks.
  - Was only the neural network used?
  - What are the parameters of cross-validation?
  - What was the size of the validation sample?
  - If possible, add the importance of the selected variables to Table 1.
- The results in Table 2 are usually presented in terms of mean and deviation in multiple samples or even for different training sessions in the same sample for neural networks. Has this been accomplished? This information is useful to support this statement in the Discussion Section: "we observed that no over-fitting occurs to impact the classification accuracy adversely".

Is the work clearly and accurately presented and does it cite the current literature?

Partly

If applicable, is the statistical analysis and its interpretation appropriate?

No

Are all the source data underlying the results available to ensure full reproducibility?

Partly

Is the study design appropriate and is the work technically sound?

Partly

Are the conclusions drawn adequately supported by the results?

Yes

Are sufficient details of methods and analysis provided to allow replication by others?

Partly

Reviewer Expertise:

Machine learning; Image processing; AI

I confirm that I have read this submission and believe that I have an appropriate level of expertise to confirm that it is of an acceptable scientific standard, however I have significant reservations, as outlined above.

## References

[1] : Diagnosis of Headaches Types Using Artificial Neural Networks and Bayesian Networks. *IEEE Latin America Transactions* .2020;18(01) : 10.1109/TLA.2020.9049462 59-66 10.1109/TLA.2020.9049462

# Peer review: Reviewer response for version 2

**DOI:** 10.5256/f1000research.28066.r64952

**Published:** 2020-08-03

**Authors:** Hugo Ordoñez Eraso

**Affiliations:** 1 Research Laboratory in Development of Software Engineering, University of San Buenaventura, Cali, Colombia; 2 Universidad de Cauca, Popayán, Colombia

The paper present an model Automatic migraine classification using artificial neural. the article presents a topic of great investigative interest, it is easy to read and its common thread is easy to follow, it is also well structured.

 To continue with its indexing process, the following considerations are recommended:

1. Improve image quality 2.
2. What hyperparameters were taken into account for the SVM model?
3. What was the value of the K neighbors that obtained the best results?
4. To better understand the results, make a graph with the data from each model.

Is the work clearly and accurately presented and does it cite the current literature?

Yes

If applicable, is the statistical analysis and its interpretation appropriate?

Yes

Are all the source data underlying the results available to ensure full reproducibility?

Yes

Is the study design appropriate and is the work technically sound?

Yes

Are the conclusions drawn adequately supported by the results?

Yes

Are sufficient details of methods and analysis provided to allow replication by others?

Yes

Reviewer Expertise:

data analyticsInformation retrievalsoftware engineering

I confirm that I have read this submission and believe that I have an appropriate level of expertise to confirm that it is of an acceptable scientific standard.

# Peer review: Reviewer response for version 1

**DOI:** 10.5256/f1000research.25589.r66038

**Published:** 2020-07-06

**Authors:** Shengyuan Yu

**Affiliations:** 1 Medical School of Chinese PLA, Beijing, China

I have carefully read and thoroughly considered your manuscript, "Automatic migraine classification using artificial neural networks".

**Our comments:**

 This is a meaningful idea to study the migraine classification by artificial neural networks. There are several significant problems with the description of the study and interpretation of the results.

 First, the preface of the article is too wordy. We need not to describe the details of migraine as a reviews, including the epidemiology, diagnosis,differential diagnosis and treatment. In the preface, we should include the brief introduction of migraine and artificial neural networks,the current status of research on this subject, the problem in this field, why we do this study.

 Second, the methods of study should include the Inclusion and exclusion criteria of the study. the reviews of classification progression of migraine should not included in this part,you can put it partial to the discussion. 

 Third, the discussion is too simple, we advice to discuss the present methods that using in the migraine classification and where is our comparative advantage, potential mechanism of the artificial neural networks using in the migraine classification. By the way, you can points out the direction of future research.

Is the work clearly and accurately presented and does it cite the current literature?

Partly

If applicable, is the statistical analysis and its interpretation appropriate?

Yes

Are all the source data underlying the results available to ensure full reproducibility?

Yes

Is the study design appropriate and is the work technically sound?

Yes

Are the conclusions drawn adequately supported by the results?

Yes

Are sufficient details of methods and analysis provided to allow replication by others?

Partly

Reviewer Expertise:

Headache

I confirm that I have read this submission and believe that I have an appropriate level of expertise to confirm that it is of an acceptable scientific standard, however I have significant reservations, as outlined above.

# Response

**Published:** 2020-07-10

**Authors:** Paola Sanchez-Sanchez

**Affiliations:** Universidad Simon Bolivar, Colombia

We appreciate the evaluation, which is very timely and pertinent. In response, and following the recommendations, a new version of the manuscript will be made

# Response

**Published:** 2020-07-13

**Authors:** Paola Sanchez-Sanchez

**Affiliations:** Universidad Simon Bolivar, Colombia

We sincerely appreciate the reviewer feedback on this work and have improved the article based on your recommendations.

 We have addressed each comment as follows in the article:

 1. We rewrite the preface focusing the content on aspects relevant to research such as a short introduction to migraine, the current state of research in the area, the problems surrounding the classification of different types of migraine, how artificial neural networks help to solve the problems and why of this study.

 2. The methods section is rewritten for better clarity in the writing. Clarifications are made regarding the inclusion and exclusion criteria, which are described in the results.

 3. The discussion section was expanded to include the validation of the results with the different models applied, the significance of the results obtained, an account of some aspects that justify the development of classification models with artificial neural networks and the comparison with some studies previous. Likewise, the conclusions was expanded to include future research fields.
